# Children’s perception on obesity and quality of life: a Mexican survey

**DOI:** 10.1186/1471-2431-14-131

**Published:** 2014-05-29

**Authors:** Mario-Enrique Rendón-Macías, Haydeé Rosas-Vargas, Miguel-Ángel Villasís-Keever, Celia Pérez-García

**Affiliations:** 1 Unidad de Investigación en Epidemiología Clínica, UMAE Hospital de Pediatría, Centro Médico Nacional Siglo XXI, Instituto Mexicano del Seguro Social, Cuauhtémoc 330 Colonia Doctores, México, D.F. CP 06720, México; 2 Unidad de Investigación Médica en Genética Humana, Centro Médico Nacional Siglo XXI, Instituto Mexicano del Seguro Social, México, D.F., México

## Abstract

**Background:**

Child obesity has become a major health problem worldwide. In order to design successful intervention strategies, it is necessary to understand how children perceive obesity and its consequences.

**Methods:**

With the aim to evaluate scholar children perception of obesity as a significant factor on the quality of life, we developed and validated the “Obesity impact on the quality of life perception-questionnaire” (ObI-Q). We surveyed 1335 healthy children aged 6–12 years, randomly selected from elementary schools in Mexico City. The ObI-Q comprises eight multiple-choice items that explore aspects related to the quality of life during adult life; such as health, life span, emotional status, lifestyle, social recognition and economic status. In order to identify perceptional modifier factors, results were analyzed through multivariable logistic regression. Variables included gender, age, and child nutritional status, as well as the child’s perception of parental nutritional status.

**Results:**

ObI-Q results showed that most children (64.71%) considered obesity as a negative condition that influences health and social performance. This perception was inversely related to age (OR = 0.64, p = 0.003), as well as to the perception of their mother nutritional status (OR = 0.47, p = 0.01).

**Conclusions:**

This study provides an overview of children’s perception on obesity and its consequences. Because the high proportion of schoolchildren who do not view obesity as an adverse consequence to the quality of life, then the results of this study could be used as part of strategies for the prevention of overweight and obesity.

## Background

Obesity has become the most prevalent nutritional disease worldwide, particularly in developing countries, where it has reached alarming numbers that multiply the medical, psychological, social and economic repercussions to face in the years to come [1-3]. Child obesity represents a special issue to focus on, since evidence points towards more premature development of obesity comorbidities formerly considered as adult diseases, such as type 2 diabetes and metabolic syndrome [4,5]. Nowadays, Mexico is one of the countries with the highest prevalence of child obesity worldwide. Data from the National Health and Nutrition Survey 2012 [6], showed a combined prevalence of children overweight and obesity of 26%, strikingly contrasted to 18.4% assessed in 1999. This increase may not be solely explained by genetic factors or population redistribution derived from migration phenomena, but also because of environmental factors linked to major changes in the quality of life, more prone to energy homeostasis imbalance and fat accumulation [7-10].

In Mexico, in the last few years, strategies designed to prevent child obesity have been carried out by using mass communication media as diffusing platform, parallel to diverse particular interventions, which mainly promote healthy nutrition and physical activity [11-14]. However, the success of these interventions is limited by the individual motivational status, which is in turn influenced by nutritional status self-perception and the attitude around obesity within each family [15,16].

On the other hand, underestimation of weight status is a common tendency, particularly in populations with high obesity prevalence. In Mexico, the erroneous but popular belief that a “chubby” child is synonymous of healthy child still prevails among medium and low income families, in which overprotective mothers commonly minimize or do not recognize the consequences of overweight and obesity. Under this perspective, children may receive distinct messages that may generate confusion and jeopardize the probability of success of any preventive intervention. Therefore, it is possible that actions or activities performed by children to prevent overweight/obesity are determined by how they perceive the impact of obesity on the physical, social or economic well-being throughout life. Currently, strategies against child obesity do not include actual perception that schoolchildren have about it. To address this need we evaluated, for the first time to our knowledge, the perception of Mexican children about the association between quality of life and obesity. Furthermore, we hypothesized that children body weight status could be a confounding variable in their responses.

## Methods

### Design and sample

We included 1,335 healthy children; 658 boys and 677 girls. They were selected from a multistep sampling conducted in 2,298 elementary schools of Mexico City. These schools are distributed along 16 territorial-political municipalities; where at the time of the survey had a total population of 762,087 students. Randomly, two schools were selected from each municipality (total = 32 schools). We estimated a minimal sample size of 810 participants, in order to obtain a normally distributed sample along the children weight spectrum according to the prevalence reported for Mexico City [6]. To ensure the representativeness and considering possible participation rate of 50%, from each school we invited 45 children of both sexes aged between 6 and 12. From 1,502 candidates, fortunately only 105 were eliminated due to one of the following reasons: they failed to obtain the consent of their parents (n = 64, 4.4%), or they did not attend the survey schedule (n = 41, 2.9%).

The Research and Ethics Committees of the Coordination of Health Research from the Mexican Social Security Institute (IMSS) approved this study. We obtained informed consent from parents and informed assent from children who agreed to participate.

### Instrument

Because we did not find a questionnaire that explores the potential impact of obesity on quality of life throughout life, we designed and developed the “Obesity impact on the quality of life perception-questionnaire” (ObI-Q). It comprises eight multiple-choice items that explore aspects related to the quality of life during adult life; such as health, life span, emotional status, lifestyle, social recognition and economic status. Each question has three possible responses: one in favor, one against and the third as doubt or inconclusive. The contents of the questionnaire were developed for three pediatricians, one psychologist and one child psychologist. They determined the aspects or domains that would be included, according to children perception. In addition, they identified the terms by which children may perceive people with obesity. In a pilot study, the OBI-Q was presented to a group of 20 children of 7–10 years old, who were asked about their understanding of the questions. The research group asked children to explain the purpose of the questions. Considering the answers, some questions were rephrased with words more appropriate for children. The modified questionnaire was tested into a new group of 10 children, and we concluded that no major changes were needed. The final version of the questionnaire was administered to 105 children aged 6 to 12 years, who were students of one school. With the results, we conducted a factorial analysis and found two domains: 1) Obesity as an ill condition (items: obesity as disease, as a risk for a chronic degenerative disease, and as factor limiting life span, lifestyle or emotional status). 2) Obesity as an obstacle to performance in life (items: socialization, economic success, and physical accomplishments). For the factorial analysis, all the items loaded from 0.54 to 0.77.

Factorial analysis yielded a Kaiser-Meyer-Olkin measure of sampling adequacy value of 0.687, and a Chi-square 121.9, 28 df Spherecity Bartlett’s Test <0.001; these values led us to conclude that the number of children surveyed was enough to accurately construct the model. Besides, the internal consistency analysis showed globally Cronbach’s alpha = 0.712. The elimination of any item did not increase the value of Cronbach’s alpha above 0.75.

### Anthropometry

Height and body weight were measured with 6 h of fasting without shoes and with light clothing by trained researchers. Height was measured with a portable stadiometer (Seca™ Model 213) with the head held in the Frankfurt plane. Children were weighted using a weighing scale (Tanita™SC-240MA) to the nearest tenth of a kilogram, minus 1 kg for clothing. BMI was calculated as kg/m^2^; children body weight status was inferred according to the International Obesity Task Force (IOTF) cut-offs for underweight, normal weight, overweight and obesity [17].

### Data collection

The physical ObI-Q was applied individually to all participants at their own school, in a private room. One of the researchers read the questions and possible answers, and children were instructed to indicate verbally the answer that best matched their thinking. Participants answered according to their own criteria, by choosing the answer that most appropriately reflected their perception of the consequences of obesity. It was emphasized that there were no right or wrong answers, and confidentiality was assured in all cases. Once the questionnaires were resolved, we obtained the anthropometric measurements. In order to evaluate body weight status perception we used the criteria used by Abalkhail et al. [18]. Children were asked to indicate how they see themselves and their parents selecting one of the following statements: “I consider myself/my mother/my father to be …” *i*) very thin, *ii)* normal, *iii*) slightly fat, *iv*) fat, which was interpreted respectively as underweight, normal weight, overweight and obese.

### Statistical analysis

Simple frequencies and relative percentages were calculated for categorical variables, whilst mean values and standard deviation were calculated for quantitative variables. The answers from the ObI-Q are presented based on the frequency of each of the eight items, without making an overall score. Analyses are described taking into account the actual nutritional status.

Difference in proportions according to body weight status was calculated through Chi-Square test. In order to identify whether there were groups of children with more favorable or less favorable perception regarding the consequences of obesity, we conducted a cluster analysis. The answers from the ObI-Q were recorded with a score of 1 to 3, in which a score of 1 was assigned to responses of negative perception, a score of 2 for a neutral perception, and a score of 3 to a favorable perception. Using a Two Step Cluster analysis classification, and by means of Bayesian Schwarz’s Criterion Clustering, two clusters were formed by grouping participants with similar responses.

We also conducted a binary logistic regression analysis to assess the effect of age, sex, and individual body weight status (actual and perceived) as well as parental body weight status on children’s perception. The analyses were performed considering the following variables: gender (dichotomous: males, females), weight status (underweight and normal, overweight, and obese), and age (6 to 8 years, 9 and 10 years, and 11 and 12 years). Odds ratio and confidence intervals at 95% were obtained. A p value < 0.05 was considered statistically significant. All the analyses were performed using SPSS version 16.0 (Chicago, Ill. USA).

## Results

### Sample characteristics

We surveyed 1335 children, 658 boys (49.3%) and 677 girls (50.7%). The mean age was 9.6 years ± 1.9 (1 SD). Overall, the results of children body weight status are shown in Table 1. A total of 599 children from both gender were either obese or overweight (44.8%), with higher frequency in boys (48.7%) than in girls (41.0%) (p = 0.005). Also, there was a higher combined prevalence of overweight and obesity relative to age, being 32.3% at 6 years and 45.7% at 12 years (p = 0.009).

**Table 1:** Distribution of body weight status

**Variable**	**Underweight (n = 84)**	**Normal weight (n = 652)**	**Overweight (n = 338)**	**Obesity (n = 261)**	**Total (n = 1335)**
	**% (n)**	**% (n)**	**% (n)**	**% (n)**	**N**
Gender*					
Boys	6.1 (40)	45.1 (297)	27.1 (178)	21.7 (143)	658
Girls	6.4 (44)	52.5 (355)	23.7 (160)	17.4 (118)	677
Age (years)**					
6–8	8.8 (37)	50.1 (211)	20.4 (86)	20.7 (87)	421
9–10	4.1 (14)	49.7 (170)	27.2 (93)	19.0 (65)	342
11–12	5.8 (33)	47.3 (271)	27.8 (159)	19.1 (109)	572
**Perception of father body weight status**^**+**^					
Underweight	5.3 (4)	3.3 (20)	4.9 (16)	7.8 (19)	59
Normal	38.1 (29)	29.1 (179)	26.1 (85)	24.6 (60)	353
Overweight	51.3 (39)	65.6 (403)	66.0 (215)	63.1 (154)	811
Obese	5.3 (4)	2.0 (12)	3.0 (10)	4.5 (11)	37
	n = 76	n = 614	n = 326	n = 244	n = 1,260
**Perception of mother body weight status**^**++**^					
Normal	4.8 (4)	4.9 (32)	4.4 (15)	3.9 (10)	61
Underweight	46.4 (39)	34.0 (220)	33.7 (114)	27.1 (70)	443
Overweight	48.8 (41)	59.1 (383)	59.2 (200)	66.3 (171)	795
Obese	0	2.0 (13)	2.7 (9)	2.7 (7)	29
	n = 84	n = 648	n = 338	n = 258	n = 1,328

Chi-Square test linear by linear, *p = 0.009, **p = 0.03.

Chi-Square test linear by linear ^+^Father p = 0.74, ^++^Mother p = 0.004.

### Obesity impact on perception of quality of life according to children body weight status

Children perception regarding obesity as a factor influencing different aspects of life was evaluated with the OBI-Q. As shown in Table 2, we found that obesity was mostly perceived (66.4%) as a disease, particularly for those who were overweight or obese (p < 0.001). Furthermore, perception of obesity in relation to life span, most of them think that there are no differences between obese and non-obese people; however, 34.6% perceived obese people as more likely to die younger than non-obese people, and this perception was more common in overweight and obese children. Another aspect perceived as negatively restricted by obesity was lifestyle, as measured trough liveliness. Although there were no significant differences between groups, results clearly showed that most children consider that obese people are not very lively (58.3%). In contrast, regarding the emotional status, most children shared the opinion that obesity is not a condition that limits socialization (56.3%) or happiness (53.1%). However, it was noted that a certain proportion of obese children (30.7%, p = 0.003) perceived that obese people have a sad life. For the rest of items that explore quality of life we did not find significant differences, although it is worth mentioning that a considerable proportion of surveyed children perceived that obese people are not sport winners (48.0%) and have limited wealth (40.5%). We did not find differences between genders.

**Table 2:** Distribution of answers to ObI-Q according to actual body weight status

**Items**	**Total % n**	**Under weight % n**	**Normal weight % n**	**Over weight % n**	**Obese 3 % n**
1. As disease*					
Obese people are sick	66.4 (887)	54.8 (46)	63.3 (413)	69.2 (234)	74.3 (194)
Obese people are healthy	33.6 (448)	45.2 (38)	36.7 (239)	30.8 (104)	25.7 (67)
2. As risk of chronic degenerative disease					
Obese people get sick more often than non-obese people	44.9 (600)	32.1 (27)	46.3 (302)	44.4 (150)	46.4 (121)
Obese people get sick as often as non-obese people	41.7(556)	44.1 (37)	39.7 (259)	44.7 (151)	41.7 (109)
Obese people get sick less often than non-obese people	13.4 (179)	23.8 (20)	14.0 (91)	10.9 (37)	11.9 (31)
3. As a conditioning factor of life span**					
Obese people die young	34.6 (462)	33.4 (28)		35.8 (121)	36.0 (94)
Obese people live as long as non-obese people	54.4 (726)	46.4 (39)	53.8 (350)	58.3 (197)	53.6 (140)
Obese people live long lives	11.0 (147)	20.2 (17)	12.6 (83)	5.9 (20)	10.4 (27)
4. As a factor associated with lifestyle					
Obese people are not very lively	58.3 (778)	54.8 (46)	58.1 (379)	60.9 (206)	56.3 (147)
Obese people are equally lively than non-obese people	31.0 (414)	32.1 (27)	32.5 (212)	27.8 (94)	31.0 (81)
Obese people are very lively	10.7 (143)	13.1 (11)	9.4 (61)	11.3 (38)	12.7 (33)
5. As a conditioning factor of emotional status***					
Obese people live sad	28.0 (374)	21.4 (18)	27.5 (179)	28.7 (97)	30.7 (80)
Obese people are equally happy than the non-obese	53.1 (709)	48.8 (41)	52.0 (339)	54.1 (183)	55.9 (149)
Obese people are happier than non-obese people	18.9 (252)	29.8 (25)	20.5 (134)	17.2 (58)	13.4 (35)
6. As an obstacle for socialization					
Obese people are solitary	27.9 (373)	23.8 (20)	29.8 (194)	26.0 (88)	27.2 (71)
Obese people have many friends as non-obese people	56.4 (752)	64.3 (54)	53.2 (347)	60.7 (205)	55.9 (146)
Obese people have more friends than non-obese people	15.7 (210)	11.9 (10)	17.0 (111)	13.3 (45)	16.9 (44)
7. As a factor for economic success					
Obese people are not very wealthy	40.5 (541)	41.7 (35)	42.0 (274)	46.2 (138)	36.0 (94)
Obese people are wealthy as non-obese people	47.0 (627)	41.7 (35)	44.8 (292)	40.8 (156)	55.2 (144)
Obese people are very wealthy	12.5 (167)	16.6 (14)	13.2 (86)	13.0 (44)	8.8 (23)
8. As a factor for physical accomplishments					
Obese people never triumph in sports	48.0 (641)	46.4 (39)	46.9 (305)	49.4 (130)	49.8 (130)
Obese people triumph in sports as much as the non-obese	41.3 (551)	41.7 (35)	41.2 (270)	41.4 (31)	40.6 (106)
Obese people often triumph in sports	10.7 (143)	11.9 (10)	11.9 (77)	9.2 (106)	9.6 (25)

Chi square test linear-by-linear, *p <0.001, **p = 0.04, ***p = 0.003.

### Perception of body weight status

Since children opinion about obesity may be influenced by their body weight self-perception, we evaluated this aspect in relation to their actual body weight status. We found that the actual body weight status and self-perception were correlated (p < 0.001), with no difference between genders (Table 3). However, it is important to mention that while children with normal weight and overweight self-perception are largely consistent with reality (67.0% and 54.1%, respectively), underweight children tend to idealize their body image; e.g., they tend to perceive themselves as normal weight children (70.2%). Something similar occurs with obese children who generally perceived themselves below the actual body weight status; mostly as overweight children (72.8%).

**Table 3:** Perception of body weight status

	**Self-perception**			
**Actual body weight status**	**Underweight % (n)**	**Normal Weight % (n)**	**Overweight % (n)**	**Obese % (n)**
**Underweight**				
Overall	13.1 (11)	70.2 (59)	16.7 (14)	0 (0)
Boys	10.0 (4)	67.5 (27)	22.5 (9)	0 (0)
Girls	15.9 (7)	72.7 (32)	11.4 (5)	0 (0)
**Normal weight**				
Overall	12.7 (83)	67.0 (437)	18.6 (121)	1.7 (11)
Boys	14.8 (44)	67.0 (199)	16.2 (48)	2.0 (6)
Girls	11.0 (39)	67.0 (238)	20.6 (73)	1.4 (5)
**Overweight**				
Overall	3.0 (10)	40.5 (137)	54.1 (183)	2.4 (8)
Boys	2.8 (5)	42.1 (75)	51.7 (92)	3.4 (6)
Girls	3.1 (5)	38.8 (62)	56.9 (91)	1.2 (2)
**Obese**				
Overall	3.4 (9)	15.3 (40)	72.8 (190)	8.5 (22)
Boys	5.6 (8)	11.9 (17)	74.1 (106)	8.4 (12)
Girls	0.8 (1)	19.5 (23)	71.2 (84)	8.5 (10)

Overall: Chi square 361.2 _9fd_ p <0.0001 (Spearman rho = 0.48).

Boys: Chi square 200.8 _9fd_ p <0.0001 (Spearman rho = 0.49).

Girls: Chi square 176.2 _9fd_ p <0.0001 (Spearman rho = 0.48).

Since there are clear precedents that demonstrate the importance of the parental influence on the behavior and opinions of their children [19,20], we asked children about their opinion on their parent’s body weight status in the same way they did for themselves. Although it was not possible to obtain real anthropometric data of parents to establish a comparison, with the gathered data we found (Table 1) that there was no relationship between children’s nutritional status and the perception of the father nutritional status (p = 0.74). As for the perception of the mother, there was a tendency to perceive them more frequently as obese by children who were classified into a group of higher BMI (p = 0.004). Thus, little more than half children with low-weight (51.2%) perceived their mothers with low or normal weight, and in no case as obese, in contrast to obese children who described their mothers mostly as overweight or obese (69.0%).

### Factors associated to obesity impact perception

By stepwise cluster analysis, children were grouped into two clusters. One in which answers showed an unfavorable perception of obesity (n = 864), and another group in which, although obesity was not perceived as favorable, it was neither perceived as different to other nutritional conditions (n = 471) (Table 4). After carrying out this categorization, a multivariate logistic analysis was performed to determine the correlation between different variables and the likelihood of an unfavorable perception on obesity. It was observed that gender does not influence the perception of children about obesity, while age was a determining factor in which the negative perception decreases inversely (OR = 0.64). Regarding the body weight status, it was observed that having a condition different to the normal weight also decreases the probability of a negative perception (underweight, OR = 0.71; obese, OR = 0.70). Conversely, the self-perception of thinness was linked to an increased probability (OR = 1.68, p = 0.03). The perception of the parent’s body weight status is also significant. Having an image of thinness of their parents also decreased the possibility of a negative perception (underweight father, OR = 0.54; underweight mother, OR = 0.47), while the image of having an obese mother made this probability to increase (OR = 1.21, p = 0.67).

**Table 4:** Variables associated to obesity perception

	**Cluster 1: Obesity as unfavorable condition**	**Cluster 2: Obesity as not unfavorable condition**	**Odds ratio (OR)**	**95% Confidence interval**	**p value**
	**(n = 864)**	**(n = 471)**			
	**% (n)**	**% (n)**			
**Gender**					
Boys (reference)	49.1 (424)	49.7 (234)	1.00	(0.8, 1.2)	0.93
Girls	50.9 (440)	50.3 (237)	0.99		
**Age (years)**					
6 to 8 (reference)	34.8 (301)	25.5 (120)			
9 to 10	25.0 (216)	26.7 (126)	0.69	(0.5, 0.9)	0.02
11 to 12	40.2 (347)	47.8 (225)	0.64	(0.5, 0.9)	0.003
**Body weight status**					
Normal (reference)	49.1 (425)	48.2 (227)			
Underweight	7.2 (62)	4.6 (22)	0.71	(0.4, 1.2)	0.22
Overweight	24.7 (213)	26.6 (125)	0.74	(0.4, 1.3)	0.31
Obese	19.0 (164)	20.6 (97)	0.70	(0.4, 1.3)	0.26
**Body weight status self-perception**					
Normal (reference)	50.7 (438)	49.9 (235)			
Underweight	9.5 (82)	6.6 (31)	1.68	(1.0, 2.7)	0.03
Overweight	36.9 (319)	40.1 (189)	0.99	(0.7, 1.3)	0.97
Obese	2.9 (25)	3.4 (16)	0.87	(0.4, 1.7)	0.71
Perception of his/her mother* (n = 1,328)					
Normal (reference)	33.8 (290)	32.5 (153)			
Underweight	3.6 (31)	6.4 (30)	0.47	(0.3, 0.8)	0.01
Overweight	60.3 (517)	59.0 (278)	1.01	(0.8, 1.3)	0.93
Obese	2.2 (19)	2.1 (10)	1.21	(0.5, 2.8)	0.67
Perception of his/her father* (n = 1,260)					
Normal (reference)	28.5 (232)	27.0 (121)			
Underweight	4.4 (36)	5.1 (23)	0.54	(0.4, 1.5)	0.53
Overweight	64.4 (523)	64.3 (288)	0.99	(0.7, 1.3)	0.94
Obese	2.7 (21)	3.6 (16)	0.69	(0.3, 1.4)	0.31

Multivariable logistic regression.

*Motherless and fatherless children were excluded from the analysis.

## Discussion

In this study we have shown that about a third of Mexican school children do not consider that obesity can adversely affect the quality of life in adulthood, which seems to be influenced by their age and their perception their mothers’ body weight status. Numerous studies have been carried out to determine factors associated with the development of obesity in children, however, to our knowledge this is the first study that focuses on the child obesity problem from their own perspective, and related to their notion on obesity consequences. At scholar age, children may not be aware of the implications of being an obese person, considering the problem outside their reality. Nevertheless, it is possible to postulate that at this stage of acquisition of values and behaviors children begin to judge adult’s lives, and to associate the results of these judgments with the nutritional status, reflected by their physical appearance. To address this question, we conducted this survey, which aim was to explore two main aspects. The first one was the perception of obesity as a disease or a condition that predisposes to increased morbidity and early mortality, while the second aspect was the perception of obesity as a limiting factor for satisfying healthy/good lives as assessed through major aspects for this particular age, such as sport and social skills. For this purpose, we designed and validated the ObI-Q, a multiple-choice questionnaire of 8 items that enabled us to examine how children perceived the relationship between obesity and adult quality of life. For the validation and reliability process of the questionnaire, we carried out the various stages suggested by Streiner and Norman [21,22]. Thus, before its application to the study population, the questionnaire was found to have both content and construct validity, and internal consistency. In addition, we conducted a pilot study to determine that the children understood the wording of the items and their answers. The ObI-Q differs from other instruments as the Youth Quality-of-Life-Weight module (YQOL-W) [23] or the Pediatric Quality of Life Inventory (PedsQL) [24], which are targeted to preadolescent and adolescent population. Besides, these instruments were designed to assess the current quality of life of individuals, so the questions are limited to situations experienced leaving out the possibility of a projection of the future of the circumstances as well as the opinion of the individual beyond himself.

Overall results showed a perception ranging from indifference to a clear view of obesity as a problem. Most children (~65%) perceived that obesity is a negative condition related to sickness and premature die. Notably, children with overweight and obesity were more aware of these complications (Table 2). This coincides with the findings of previous studies with adolescent populations, in which an inverse relationship between weight status and health-related quality of life was detected [25], and Jalali-Farahani S, Chin YS, Amiri P, Mohd Taib MN: **Body mass index (BMI)-for-age and health-related quality of life (HRQOL) among high school students in Tehran.***Child Care Health Dev* 2013. doi:10.1111/cch.12103, forthcoming]. Our results show that the negative perception of obesity probably originates from an early life stage, and most children are aware of the consequences of obesity in relation to health, especially those who experience it in their own person and/or through the close mother figure. However, there was still a large proportion not sensitized about it, thus, it will be important to explore the factors that influence the formation of this view and direct it properly as a support for preventive strategies, as previously suggested [26].

Regarding the children perception of obesity on physical activity and success, it was clear that most children have realized that obesity creates limitations on physical performance, which has been shown to be functionally accurate, independent of gender, ethnicity, and age [27].

On the other hand, the cluster analysis show that the views of children of school age about the adverse consequences of obesity are not associated with neither actual nutritional status nor self-perception of it. This circumstance is probably determined by the perception of body shape, which is developed during adolescence [28].

In contrast with this negative perception, the results showed that the emotional area was not perceived as affected as the physical. In this regard, previous results from different analyzes of the psychological and depressive symptoms relative to the weight are contradictory [29-32], however, there is substantial heterogeneity between ethnicities and age groups of these studies that may explain these differences. In the particular case of Mexican population, we can argue that overweight and obesity have become very common conditions, to the extent that affected individuals are not excluded from the circles of daily living, and emotional development is not conditioned by their weight, which is positively perceived by children. This idea is reinforced by our own results, which show that obese children perceive their mothers mostly as overweight, opening the possibility that these children build their perception around the mother figure, traditionally protective and loving. This set of circumstances may skew the child perception towards a positive opinion about obesity and overweight, as has been recently demonstrated in a Mexican-American population [33]. Such observation has social and psychological connotations that do not concern this study, but needs to be further analyzed. In the meantime, the emotional aspect should continue to be considered invariably in the design of intervention strategies against obesity, since it represents a key element of their success.

Despite the strengths of this study, we recognize its limitations. For reasons of feasibility, measuring the parents’ body weight status was not performed, so we considered children perception. In addition, we surveyed only urban children where the problem of obesity is very high; therefore, it may be necessary to explore this phenomenon in populations of different socioeconomic status, as well as in rural environment. It may also be appropriate to analyze the evolution of this perception over time.

## Conclusion

This study provides an overview of children’s perception on obesity and its consequences. Because the high proportion of schoolchildren who do not view obesity as an adverse consequence to the quality of life, then the results of this study could be used as part of strategies for the prevention of overweight and obesity.

## Competing interests

The authors declared that they have no competing interests.

## Authors’ contributions

RM participated in the conception and design of the study, also participated in the field camp for data acquisition, and performed the statistical analysis. RV participated in the drafting of the manuscript and revising critically. VK participated the design of the study and drafting and revising critically the manuscript. GP participated in the design of the study and acquisition of data. All authors read and approved the final manuscript.

## Pre-publication history

The pre-publication history for this paper can be accessed here:

http://www.biomedcentral.com/1471-2431/14/131/prepub
